# Genome-Wide Identification and Expression Analysis of the S-Locus-Specific Glycoprotein/EP1 Gene Family in Cotton (*Gossypium hirsutum* L.)

**DOI:** 10.3390/ijms27209303

**Published:** 2026-10-20

**Authors:** Conghua Feng, Yi Liu, Suen Liu, Junyi Geng, Hui Sun, Dan Li, Ruixuan Liu, Yuyuan Qian, Baosheng Guo

**Affiliations:** Key Laboratory of Cotton Biology and Genetic Breeding in Huanghuaihai Semiarid Area, Ministry of Agriculture and Rural Affairs, Hebei Key Laboratory of Cotton Bio-Breeding and Cultivation Physiology, Shijiazhuang 050051, China; fch19942024@163.com (C.F.); liuyi1105@126.com (Y.L.); liusuen2026@126.com (S.L.); gengjunyi66@126.com (J.G.); 13832308563@163.com (H.S.); lidan5279@163.com (D.L.); liuruixuan213@163.com (R.L.)

**Keywords:** *Gossypium hirsutum*, *GhEP1* gene family, drought tolerance, transcriptomic analysis, abiotic stress, gene expression

## Abstract

The S-locus-specific glycoprotein/EP1 gene family encodes a class of epidermis-specific secretory glycoproteins involved in plant responses to abiotic stress. However, their role in cotton (*Gossypium hirsutum*) drought response remains unclear. Here, we identified 12 *GhEP1* genes from the *G. hirsutum* genome, clustered into three subfamilies. Structural predictions indicated that most GhEP1 proteins are hydrophilic and localised to the outer side of the plasma membrane or nucleus, with a few containing transmembrane domains. Collinearity analysis revealed that segmental and tandem duplications drove family expansion, with a high degree of collinearity conservation among dicotyledonous plants. Transcriptomic data showed generally low *GhEP1* expression across tissues, but *GhEP1-2*, *GhEP1-6*, *GhEP1-7* and *GhEP1-8* responded significantly to NaCl, PEG-induced drought and cold stresses. qRT-PCR further confirmed that the expression of most *GhEP1* genes was significantly up-regulated in roots under drought stress, while *GhEP1-3* was induced in leaves, indicating tissue-specific regulatory divergence. This study systematically characterises the *GhEP1* family and provides potential genetic resources for improving drought tolerance in cotton.

## 1. Introduction

Cotton is the world’s most important source of natural textile fibres and occupies a key position in the agricultural and economic landscape [1]. However, its production is severely constrained by abiotic stresses such as drought and salinity, with drought stress having the most pronounced impact [2]. The flower-boll stage is the period during which cotton is most sensitive to water demand; if drought occurs during this phase, it directly inhibits plant growth, triggers massive shedding of buds and bolls, and leads to a decline in fibre quality and reduced disease resistance, ultimately resulting in a dual loss of yield and quality [3,4]. This issue has become a core bottleneck constraining the high-quality development and self-reliance of China’s cotton industry. At present, molecular breeding through the identification of drought-tolerant genes represents the fundamental approach to addressing this challenge and enhancing the sector’s climate resilience [5]. Against the backdrop of increasingly severe global climate change, accelerating the identification of drought-tolerant genes has become an urgent strategic task for ensuring the stability and sustainability of global cotton production [6,7].

Drought stress is the primary abiotic factor limiting crop yield and quality. Global climate models predict that arable land will face more frequent extreme drought events, whilst traditional drought-tolerant breeding progresses slowly due to its reliance on phenotypic selection [8,9,10]. With the rapid development of functional genomics, research into plant drought tolerance is entering a new phase characterised by a four-dimensional analysis encompassing ‘genes-networks-phenotypes-environment’ [11,12]. Drought stress induces plants to initiate a coordinated morphological response characterised by ‘deep root penetration, leaf curling, shortening and thickening of the stem, and forward displacement of reproductive organs’ [13,14]. By reducing transpiration, optimising water capture and accelerating generational turnover, this response achieves a survival-reproduction trade-off under arid conditions and serves as a key visual indicator of drought tolerance [15,16]. In response to drought stress, plants initiate a multi-level, coordinated response programme ranging from signal perception to whole-plant protection [17]. Research over the past decade has established a central signalling cascade centred on ABA-*SnRK2*-*OST1*: a decline in soil water potential triggers a dual Ca^2+^-ROS pulse at the root tip; upon binding to ABA, the *PYR*/*PYL* receptors lift the inhibition of *PP2C*, thereby activating the autophosphorylation of *SnRK2.6*/*OST1* [18,19]. Once activated, *OST1* exerts protective effects via two parallel pathways: firstly, it phosphorylates *SLAC1*/*SLAH3* and *KAT1*, driving K^+^ efflux from guard cells and a reduction in turgor pressure; within 5–10 min, stomatal aperture decreases by 40–60 per cent, achieving ‘immediate protection against water loss’; secondly, it phosphorylates the *AREB*/*ABF* transcription factors, initiating the expression of downstream *LEA*, dehydrogenase (DHN) and osmoregulatory enzyme genes, thereby laying the molecular foundation for ‘long-term turgor maintenance’. At the cellular level, this central signalling pathway is coupled with the ‘osmotic-antioxidant-hydraulic’ triple defence line, forming quantifiable physiological indicators [20]. By focusing on the ‘ABA-*SnRK2*-*OST1*’ node and integrating stomatal behaviour, osmotic regulation, antioxidant efficiency and hydraulic recovery rates, a ‘Drought Resistance Index (DRI)’ can be constructed for use in early-stage screening and the evaluation of gene-editing outcomes in molecular design breeding [21,22,23]. These ABA-centred mechanisms operate alongside ABA-independent pathways, in which *DREB* transcription factors bind DRE/CRT elements to activate stress-responsive genes, and are further complemented by broader regulatory layers including *NAC*/*MYB*/*WRKY* transcription factor networks, osmotic adjustment through compatible solute accumulation, and Ca^2+^- and ROS-mediated early signalling events [24,25]. Together, these interconnected modules constitute the molecular framework underlying plant drought adaptation.

Secreted proteins serve as key mediators for the exchange of information, substances and energy between plants and their environment, and play a vital role in regulating a wide range of physiological processes [26]. Research indicates that secreted proteins are involved in biological processes such as cell wall assembly, intercellular communication, signal transduction, responses to environmental stimuli, and defence against pathogens [27,28]. Under environmental stress, plant varieties with higher resistance often exhibit increased secretion of cell-surface glycoproteins, forming thicker layered structures; whereas in susceptible varieties, glycoproteins are reduced or even shed [29]. Hydroxyproline-rich glycoproteins (HRGPs) are among the most abundant structural proteins in the cell wall, primarily comprising extensins, arabinogalactan proteins (AGPs), proline- and hydroxyproline-rich proteins, and lectins [30]. Among these, the function of the epidermal-like secretory glycoprotein EP1-like remains unclear. Engelen et al., were the first to clone the *EP1* gene from carrots; this gene shares homology with the Brassica S-locus glycoprotein (SLG) but does not participate in the self-incompatibility response. Researchers speculate that it may influence water transport via specific regulatory pathways [31]. A comparison with the UniProt database shows that *Arabidopsis* EP1-like glycoprotein 3 (Q9ZVA4) and EP1-like glycoprotein 4 (Q9ZVA5) are both localised to the cell wall; GO functional annotations suggest that both may be involved in carbohydrate binding [32].

In this study, we aimed to systematically identify and characterize the *GhEP1* genes from the *Gossypium hirsutum* genome. Through HMMER-based searches, a total of 12 candidate *GhEP1* genes were identified. Their chromosomal localization, gene amplification, gene structure, and upstream promoter cis-acting elements were systematically predicted and analyzed. In addition, the physicochemical properties, subcellular localization, conserved motifs, and phylogenetic relationships of the encoded proteins were characterized. To identify *GhEP1* members potentially involved in drought stress responses, we analyzed the transcriptomic expression profiles and qRT-PCR results of the representative gene *GhEP1*. This study represents the first comprehensive analysis of the *GhEP1* genes integrating genomics, transcriptomics, and qRT-PCR approaches, leading to the identification of *GhEP1* as a candidate gene involved in drought response. Based on the *G. hirsutum* TM-1 reference genome (v2.1) and the COTTONOMICS multi-omics database, we performed a genome-wide identification and functional annotation of the *GhEP1* family, providing a theoretical foundation for further exploration of key regulatory genes involved in drought stress responses in cotton.

## 2. Results

### 2.1. Identification of the GhEP1 Gene Family and the Physicochemical Properties of Its Proteins

To systematically elucidate the function of the *GhEP1* gene family in *G. hirsutum*, we identified and catalogued the members of this family. After removing redundancies and incorporating NCBI chromosome localisation data, we ultimately identified 12 *GhEP1* genes (Supplementary Table S1). Analysis of the physicochemical properties of the family proteins revealed that *GhEP1s* encode between 177 and 459 amino acids, with an average of 387.7 amino acids; their molecular weights were generally greater than 40 kDa (accounting for 75 per cent). The isoelectric point ranged from 5.93 to 9.02, with 7 being acidic proteins and 5 being basic proteins. Eleven members had an instability index of less than 40, indicating high stability (with the exception of *GhEP1-11*); all 12 proteins were found to be hydrophilic. Subcellular localisation predictions indicated that all 12 GhEP1 proteins are localised to the cell membrane or the nucleus.

Chromosomal localisation results show that the 12 *GhEP1* genes are distributed across six chromosomes in *G. hirsutum* (Figure 1). Of these, chromosomes D01 and D10 harbour the largest number of members (three), whilst the remaining chromosomes contain one or two genes. Four *GhEP1* tandem repeat clusters (*GhEP1-1*/*GhEP1-2*, *GhEP1-4*/*GhEP1-5*, *GhEP1-6*/*GhEP1-7*/*GhEP1-8* and *GhEP1-9*/*GhEP1-10*/*GhEP1-11*) were detected on chromosomes A01, A13, D01 and D10.

### 2.2. Phylogenetic Analysis and Sequence Alignment of the GhEP1 Gene Family

A phylogenetic tree was constructed using the Neighbour-Joining (NJ) method in MEGA12 (v12.1.2), based on the 38 EP1 amino acid sequences. The phylogenetic analysis divided the *GhEP1* family into three distinct clades; whilst the number of members varied between clades, the sequences within each clade exhibited a high degree of homology (Figure 2). *G. hirsutum* is represented only in Group I and Group III, with two members in Group I and ten in Group III.

To further validate conservation, transmembrane analysis and tertiary structure prediction were performed on 12 GhEP1 proteins. The results showed that some EP1 proteins (GhEP1-1; GhEP1-3; GhEP1-6) contain typical transmembrane helix regions, and the tertiary structure analyses of most GhEP1-1 proteins exhibit similarities (Figures S1A–L and S2A–L). In summary, the *GhEP1* gene family is relatively conserved throughout evolution.

### 2.3. Analysis of Motifs, Conserved Domains and Gene Structure in the GhEP1 Gene Family

To elucidate the evolutionary relationships within the *GhEP1* gene family, we conducted a multidimensional analysis integrating phylogenetic trees, conserved motifs, protein domains and gene structure (Figure 3A–D). A total of eight conserved motifs were identified across the 12 GhEP1 protein sequences. Whilst there were variations in the number and distribution of motifs, the family as a whole exhibited a high degree of conservation: all members contained at least four motifs, with Motif-7 present in all 12 members, constituting a characteristic hallmark of the EP1 domain (Figure 3B, Table S2). Domain analysis revealed that GhEP1 proteins on the same branch of the phylogenetic tree share a high degree of conservation (Figure 3C). In terms of gene structure, eight *GhEP1* genes are single-exon genes, whilst the remaining four members contain one intron (Figure 3D).

### 2.4. Collinearity Analysis of the GhEP1 Gene Family

Analysis of intraspecific collinearity further identified eight *GhEP1* fragment duplication events (Figure 4). The Ka/Ks ratios for all *GhEP1* gene pairs are less than 1 (Table S3). To elucidate interspecies evolutionary relationships, *G. hirsutum* was compared for colinearity with *Arabidopsis thaliana*; *Cucumis sativus*; *Theobroma cacao*; *Medicago sativa*; *Brassica napus*; *Populus trichocarpa*; *Malus domestica*, yielding 5, 6, 4, 1, 5, 15 and 11 pairs of homologous genes, respectively (Figure 5A–G). The results indicate that the *GhEP1* family is evolutionarily more closely related to woody plants.

### 2.5. Analysis of Cis-Acting Elements in the Promoters of the GhEP1 Gene Family

Promoter cis-acting elements are involved in the growth and development, stress response and phytohormone response of *G. hirsutum*. Stress-related cis-acting elements (such as MYB, MYC and ARE) are enriched in the majority of *GhEP1* genes; these *GhEP1* genes (e.g., *GhEP1-5* and *GhEP1-9*) may play a key role in responding to adverse environmental conditions. Furthermore, the promoters of the *GhEP1-2*, *GhEP1-6* and *GhEP1-7* genes are enriched with ABRE (Abscisic Acid Response Element) cis-acting elements, suggesting they may be responsive to the hormone abscisic acid. The promoters of *GhEP1-6* and *GhEP1-7* contain a large number of promoter elements associated with growth and development, such as the G-box (Figure 6).

### 2.6. Analysis of the Expression Patterns of the GhEP1 Gene Family

To elucidate the functions of the *GhEP1* gene family in cotton growth and development, as well as in responses to abiotic stress, this study utilised heatmaps to visualise the transcriptomic expression profiles of the *GhEP1* gene family in different tissues of *G. hirsutum* under various abiotic stress conditions (Table S4, Figure 7A,B). The tissue expression heatmap revealed that the vast majority of *GhEP1* gene family members were barely expressed in the eight different tissues; however, *GhEP1-8* maintained high expression levels in cotton petals and stamens, suggesting that *GhEP1-8* may play an important regulatory role in the formation of cotton petals and stamens (Figure 7A). The stress expression heatmap shows that *GhEP1-2* and *GhEP1-6* were significantly up-regulated under cold stress, and that *GhEP1-2* exhibited increased expression under NaCl stress (Figure 7B). Under drought stress simulated by 200 mM mannitol, *GhEP1-2*, *GhEP1-7* and *GhEP1-8* were significantly up-regulated in the roots, and *GhEP1-7* was also up-regulated in the leaves following drought stress. To summarise the drought-responsive expression changes, a heatmap was generated showing the log^2^ fold changes in *GhEP1* genes in roots and leaves under drought stress relative to the control (Figure 7C).

qRT-PCR analysis revealed that members of the *GhEP1* gene family exhibited distinct expression patterns in different tissues under drought stress in cotton. Following drought stress treatment, the expression levels of *GhEP1s* in roots were significantly higher than those in leaves (Figure 8 and Figure 9). After drought stress treatment, the expression of the vast majority of *GhEP1s* was up-regulated in roots, whilst *GhEP1-5* was initially up-regulated and subsequently down-regulated to normal levels. *GhEP1-7* was initially downregulated but rapidly upregulated after 16 h (Figure 8). In leaves, the changes in *GhEP1* expression were relatively minor, with *GhEP1-5* and *GhEP1-9* being downregulated. In leaves, *GhEP1-3* was significantly upregulated following drought stress (Figure 9).

## 3. Discussion

Drought is one of the most significant abiotic stress factors affecting cotton production, profoundly influencing multiple aspects of plant growth and development [33]. As a class of epidermis-specific secretory glycoproteins localised to the cell wall, the function of S-locus-specific glycoprotein/EP1 in plant stress responses has long been overlooked, and its biological role remains only partially understood [34]. Through the systematic characterisation of the *GhEP1* gene family in *G. hirsutum*, this study has revealed multiple patterns regarding the family’s structural characteristics, evolutionary trajectory and expression regulation, thereby providing a new perspective on understanding the potential mechanisms by which secreted proteins contribute to drought adaptation.

Within the vast system of plant secreted proteins, EP1 may, owing to its unique structural characteristics, be a potential participant in stress responses [35]. Structural predictions from this study indicate that all 12 GhEP1 proteins are hydrophilic; among them, GhEP1-1, GhEP1-3 and GhEP1-6 contain typical transmembrane helix regions (Figure S1), suggesting that they may be localised on the outer side of the plasma membrane in a membrane-anchored conformation. This topological structure provides the physical basis for them to sense changes in the extracellular environment. Subcellular localisation predictions further indicate that all GhEP1 proteins are localised to the cell membrane or the nucleus. More importantly, tertiary structure predictions reveal significant conformational differences between transmembrane and non-transmembrane members (Figure S2), suggesting that a functional division of labour may exist within the family: transmembrane members may primarily undertake signal sensing functions, whilst non-transmembrane members may be directly involved in the modification of cell wall components or be released as signalling molecules into the extracellular space. This structure–function coupling provides a structural entry point for understanding how EP1 translates extracellular stress signals into intracellular responses [36].

From an evolutionary perspective, phylogenetic analysis divides the *GhEP1* family into three subfamilies, with *G. hirsutum* members predominantly distributed across Group I and Group III (Figure 2). Group III comprises 10 members, indicating that this subfamily has undergone significant expansion in cotton. Analysis of conserved motifs identified eight conserved motifs, of which Motif-7 is present in all 12 members and constitutes a characteristic hallmark of the EP1 domain (Figure 3B, Table S2). Gene structure analysis revealed that eight *GhEP1* genes are single-exon genes, whilst the remaining four members contain a single intron (Figure 3D). Interspecific colinearity analysis indicated that the *GhEP1* family retained 15 and 11 homologous gene pairs with *P*. *trichocarpa* and apple, respectively, whereas only 5 pairs remained in the model plant *A*. *thaliana* (Figure 5); this distribution pattern suggests that the *EP1* family may have been subject to stronger selective pressure during the adaptive evolution of perennial woody plants [37]. Woody plants are exposed to fluctuating soil moisture conditions over long periods; the need to optimise water transport efficiency in their root architecture and vascular system may have exerted a directional influence on the evolution of cell wall-localised proteins such as those in the *EP1* family [38,39]. Intraspecific colinearity analysis further identified eight segmental duplication events, and the Ka/Ks ratios for all *GhEP1* gene pairs were less than 1 (Figure 4, Table S3), indicating that this family has been primarily driven by purifying selection during evolution and exhibits a high degree of functional conservation [40].

At the regulatory level, analysis of promoter cis-acting elements revealed that the widespread distribution of ABRE elements closely links *GhEP1* to the ABA signalling pathway (Figure 6). As a key hormone in the drought response, ABA signals are transmitted via the *SnRK2*-*OST1* kinase cascade to downstream transcriptional networks, ultimately driving a series of physiological adaptation responses [41,42]. ABRE cis-acting elements are enriched in the promoters of *GhEP1-2*, *GhEP1-6* and *GhEP1-7* (Figure 6), suggesting that these members may be located within functional modules at the output end of the ABA signalling pathway, participating in the execution of ABA-initiated cell wall remodelling or water retention strategies. Furthermore, stress-associated cis-acting elements such as MYB and MYC are enriched in most *GhEP1* members (Figure 6), further supporting the family’s potential involvement in abiotic stress responses. Notably, this pattern of promoter element configuration suggests that *EP1* may achieve rapid transcriptional responses following drought perception via ABRE-dependent transcriptional regulatory pathways [43]. Notably, overexpression of *GhA01EP1* (corresponding to *GhEP1-3* in this study) enhanced drought tolerance in *Arabidopsis* through hormone metabolism and antioxidant pathways, implying that *EP1* members may act downstream of ABA/JA signaling [34]. This is consistent with the ABRE enrichment in several *GhEP1* promoters and supports a model in which hormonal cues are translated into cell wall reinforcement and ROS homeostasis under drought.

The tissue-specific expression patterns further reveal the potential sites of action for *EP1*. The transcriptomic heatmap shows that the vast majority of *GhEP1* members are scarcely expressed in eight different tissues, whereas *GhEP1-8* maintains high expression levels in cotton petals and stamens (Figure 7A), suggesting that *GhEP1-8* may play a specific regulatory role in the development of cotton floral organs. The high-abundance expression of this family in floral organs and roots, rather than its predominant expression in leaves-the primary organ of photosynthesis-suggests that its function may be more oriented towards structural support and substance transport than towards metabolic activities; this distribution pattern is highly consistent with the biochemical properties of EP1 as a cell wall protein. Under abiotic stress, *GhEP1-2* and *GhEP1-6* were significantly up-regulated under cold stress, whilst *GhEP1-2* showed increased expression under NaCl stress (Figure 7B). Under drought stress simulated by mannitol, *GhEP1-2*, *GhEP1-7* and *GhEP1-8* were significantly up-regulated in the roots, whilst *GhEP1-7* was also up-regulated in the leaves (Figure 7B). qRT-PCR further confirmed that, following drought stress, the expression of the vast majority of *GhEP1* genes was significantly up-regulated in the roots, whilst changes in the leaves were relatively minor (Figure 8 and Figure 9). *GhEP1-5* exhibited an expression profile in roots characterised by an initial upregulation followed by a return to baseline levels, whilst *GhEP1-7* was rapidly upregulated after 16 h (Figure 8). Most members displayed consistent time-dependent upregulation patterns, although a few members (e.g., *GhEP1-5* and *GhEP1-9* in leaves, and *GhEP1-7* initially in roots) exhibited downregulation at specific time points or in specific tissues; this dynamic characteristic suggests that the family is not merely a ‘bystander’ in the stress response, but is involved in a continuous response process spanning from the early stages of stress perception to the later stages of physiological adaptation. This pattern of sustained expression increase contrasts with the ‘pulse-like’ upregulation characteristic of transient emergency responses, suggesting that *EP1* may perform sustained functions in cell wall reinforcement, lignin deposition, or the maintenance of transport channels for osmoregulatory substances. Following drought perception, ABA signalling drives the upregulation of specific *GhEP1* members via ABRE elements; these secreted proteins may, on the one hand, help clear toxic aldehydes generated by stress by enhancing the activity of the acetaldehyde dehydrogenase system, and on the other hand, participate in regulating the activity of the lignin synthesis pathway, promoting vascular wall thickening to maintain water conduction efficiency [39]. This dual function of ‘metabolic detoxification’ and ‘structural reinforcement’ corresponds precisely to the two core strategies of plant drought adaptation: damage control and resource conservation. *GhEP1-7* respond to drought stress in both roots and leaves, whilst *GhEP1-2* and *GhEP1-8* are specifically up-regulated in roots (Figure 7B and Figure 8). This tissue-specific response pattern suggests that functional differentiation has occurred within the family, and that different members may perform distinct drought adaptation functions in different tissues.

Although the present study has conducted a comprehensive genomic survey of the *GhEP1* family, several gaps remain that require urgent attention. Structural predictions indicate that some members contain transmembrane helices; however, the specific topological arrangement of EP1 proteins on the cell membrane and the nature of their physical interactions with cell wall components remain unclear. Collinearity analysis reveals that the *GhEP1* family shares a large number of homologous gene pairs with woody plants, suggesting that functional studies should draw more heavily on woody plants rather than relying solely on herbaceous model organisms such as *Arabidopsis*. Furthermore, the degree of functional redundancy and the specificity of functional division among members of this family still need to be distinguished through analyses of mutant phenotypes and tissue-specific expression. Future research should focus on: mapping the molecular functional landscape of the *GhEP1* family under drought stress by combining phosphoproteomics with cell wall component analysis; validating the functions of candidate genes such as *GhEP1-2*, *GhEP1-7* and *GhEP1-8* using VIGS or CRISPR/Cas9 technology; and evaluating their potential application in the genetic improvement of cotton for drought tolerance through overexpression or gene editing approaches. Although this study was restricted to *G. hirsutum* and functional validation of *GhEP1-3* is still ongoing in T1 transgenic lines, the drought-responsive *GhEP1* genes identified here serve as promising candidates for future functional characterization. In addition, future studies should incorporate time-matched mock controls and field-based drought trials to validate the drought-responsive candidates under natural conditions, and to evaluate their potential in cotton breeding programs. Although functional validation (e.g., VIGS or stable transgenic analysis) was not included because the transgenic lines are currently at the T1 generation and require further self-pollination to obtain homozygous lines, the drought-responsive *GhEP1* genes identified here should be regarded as promising candidates for future functional characterization.

## 4. Materials and Methods

### 4.1. Test Materials

The cotton material used in this study was the *JIN668* line. *JIN668* was provided by the Cotton Research Institute of the Chinese Academy of Agricultural Sciences and is a commonly used model recipient in cotton genetic transformation research. After soaking the cotton seeds in water for 14 h, they were sown in soil (a 1:1 mixture of potting compost and vermiculite). Two months later, cotton plants with consistent growth characteristics were selected and subjected to a drought-simulation treatment using D-mannitol (200 mM); leaves, stems and roots were then collected for RNA extraction, and the resulting RNA was used for quantitative PCR (qPCR) and transcriptomic sequencing analyses [44].

### 4.2. Identification of the GhEP1 Gene Family in G. hirsutum and Analysis of the Properties of Its Proteins

The *G. hirsutum* TM-1 reference genome (v2.1), transcripts, amino acid sequences and the 2000 bp upstream promoter sequences were downloaded from the COTTONOMICS database (http://cotton.zju.edu.cn/, accessed on 2 November 2025) [45]. The S-locus-specific glycoprotein/EP1 domain (PIRSF002686) was retrieved from the Phytozome 14.0 database (https://phytozome-next.jgi.doe.gov/, accessed on 25 October 2025) and used as a query to search the *G. hirsutum* protein database using HMMER v3.4 (University of Washington, Seattle, WA, USA) with an E-value threshold of 1 × 10^−5^. All hits were further validated by BLASTP against the NCBI non-redundant protein database (E-value < 1 × 10^−5^), and redundant sequences were manually removed. Finally, 12 non-redundant *GhEP1* genes were identified and named *GhEP1-1* to *GhEP1-12* according to their chromosomal locations. The *G. hirsutum* TM-1 reference genome (v2.1), transcripts, amino acid sequences and the 2000 base pairs upstream of the ATG promoter region were extracted from the COTTONOMICS database (http://cotton.zju.edu.cn/, accessed on 2 November 2025) [46]. *GhEP1s* were named according to their chromosomal location (*GhEP1-1* to *GhEP1-12*). The locations of the *GhEP1* genes and chromosome sizes were obtained from the COTTONOMICS database and visualised using the TBtools-II (v2.390) software (South China Agricultural University, Guangzhou, Guangdong, China) [47]. The physicochemical properties of the proteins were predicted using the ProtParam website (https://web.expasy.org/protparam/, accessed on 5 November 2025). Subcellular localisation predictions for the *GhEP1* gene family were carried out using the Plant-mPLoc website (http://www.csbio.sjtu.edu.cn/bioinf/plant-multi/, accessed on 8 November 2025) [48].

### 4.3. Sequence Alignment and Phylogenetic Analysis of the GhEP1 Gene Family

Using the MEGA12 (v12.1.2) software, a phylogenetic tree of the *GhEP1* gene family was constructed using the Neighbour-Joining (NJ) method, and the reliability of the branches was assessed by 1000 bootstrap resamples [49]. The resulting Newick file was subsequently imported into the iTOL website (https://itol.embl.de/, accessed on 12 November 2025) for advanced visualisation, in order to clearly illustrate the evolutionary relationships [50]. To investigate the evolutionary relationships of *GhEP1* genes with those from other plant species, we selected representative EP1 protein sequences from four species: *A. thaliana* (model dicotyledon), *O. sativa* and *Z. mays* (monocotyledon representatives), and *M. domestica* (woody dicotyledon showing high collinearity with cotton). A total of 38 EP1 protein sequences (including 12 GhEP1 proteins) were used for phylogenetic tree construction.

### 4.4. Analysis of Conserved Motifs, Conserved Domains and Gene Structure in the GhEP1 Gene Family

To analyse the structural characteristics of the *GhEP1* gene family, we first imported a file containing all *GhEP1* gene IDs and the gff3 annotation file into TBtools-II (v2.390) to visualise their exon-intron arrangements. Subsequently, the GhEP1 protein sequences were submitted to MEME Suite (https://meme-suite.org/meme/index.html, accessed on 14 November 2025); whilst retaining the default parameters, the number of motifs was set to 8 to generate the corresponding MAST results. The resulting protein sequences were also uploaded to the NCBI protein database (https://www.ncbi.nlm.nih.gov/Structure/bwrpsb/bwrpsb.cgi, accessed on 15 November 2025) to obtain the Hitdata file. Finally, the three types of data mentioned above were integrated into the TBtools-II (v2.390) platform to jointly construct a phylogenetic tree, a map of conserved motifs and a gene structure diagram for the *GhEP1* family, thereby enabling a systematic analysis of this family at the sequence, evolutionary and structural levels [51].

### 4.5. Collinearity Analysis of the GhEP1 Gene Family

MCScanX analysis was performed on the FAST and GFF3 files of the *G. hirsutum* genome using TBtools-II (v2.390) with default parameters. We further assessed the collinearity between *G. hirsutum* and other species [52]. We used the default parameters of the One Step MCScanX function in the TBtools-II (v2.390) software to identify potential homologous genes. We compared the FAST and GFF3 files of the *G. hirsutum* genome with those of other species and obtained three key files: the genomic GFF file, the CTL file (control file) and the Collinearity file. First, we manually removed unnamed chloroplast and mitochondrial sequences from the CTL file, reordered the sequences, and finally visualised the results using TBtools-II (v2.390).

### 4.6. Analysis of Cis-Acting Elements in the Promoters of the GhEP1 Gene Family

Transcriptional regulatory elements within the 2000 bp promoter region upstream of the *GhEP1* gene family were predicted using the PlantCARE website (http://bioinformatics.psb.ugent.be/webtools/plantcare/html/, accessed on 20 November 2025), and the results were visualised using TBtools-II (v2.390) and Excel (Microsoft Office 2022, Microsoft Corporation, Redmond, Washington, USA) [53].

### 4.7. Transcriptomic Analysis of the GhEP1 Gene Family

To systematically analyse the expression characteristics of the *GhEP1* gene family, this study downloaded the corresponding transcriptomic dataset from the COTTONOMICS public database (http://cotton.zju.edu.cn/index.htm, accessed on 22 November 2025). This dataset comprises FPKM expression matrices for various tissues of *G. hirsutum* (roots, stems, leaves, etc.) as well as samples subjected to abiotic stress treatments (salinity, simulated drought, and cold). These datasets are available under the accession number CNP0002656. Subsequently, expression heatmaps were generated using the ‘HeatMap’ module of the TBtools-II (v2.390) software. Row-scale normalisation was applied, whilst the remaining parameters were left at their default settings, to visually illustrate the differences in the expression profiles of *GhEP1s* across different tissues and under various stress conditions [54].

### 4.8. Quantitative Real-Time (qRT-PCR) Analysis of the GhEP1 Gene Family

To analyse the expression patterns of the *GhEP1* genes under drought stress, total RNA was extracted from drought-treated samples using the Total RNA Extraction Kit ((Cat: R1200, Beijing Solarbio Science & Technology Co., Ltd., Beijing, China)); first-strand cDNA was subsequently synthesised using the Hifair^®^ V Reverse Transcriptase (Cat No.11300ES92; Yeasen, Shanghai, China). qRT-PCR was performed using the 5×SNP Genotyping Lyo qPCR MIX (CW3398S, ComWin Biotech, Beijing, China) following the manufacturer’s protocol. Specific primers were designed using the NCBI Primer-BLAST online tool (https://www.ncbi.nlm.nih.gov/tools/primer-blast/index.cgi?LINK_LOC=BlastHome, based on BLAST+ v2.14.0, accessed on 28 November 2025) (Table S5). qRT-PCR was performed on a CFX96 Touch™ real-time quantitative PCR system (Bio-Rad, USA) using the SYBR Green detection system. Reaction programme: 15 min pre-denaturation at 95 °C; 35 cycles of 10 s denaturation at 95 °C, 30 s annealing at 58 °C, and 30 s extension at 72 °C. Relative expression levels were calculated using the 2^−∆∆CT^ method, with *GhUBQ7* (*Ghir_D12G021700*) serving as the internal reference gene [55].

### 4.9. Data Analysis

We analysed the qRT-PCR experimental data using GraphPad Prism (v9.0) (GraphPad Software, Boston, MA, USA) [51]. For relative gene expression levels, we performed a one-way analysis of variance (ANOVA) with Bonferroni multiple comparison tests (*p* < 0.05). Prior to performing the ANOVA, the data were tested for normality and homogeneity of variances.

## 5. Conclusions

In this study, we performed a genome-wide identification and characterization of the *GhEP1* gene family in *G. hirsutum*, identifying a total of 12 *GhEP1* genes distributed across six chromosomes and classified into three subfamilies. Structural and evolutionary analyses revealed that the *GhEP1* family is relatively conserved, with segmental and tandem duplications driving its expansion and purifying selection maintaining its functional stability. Promoter analysis indicated a closer evolutionary relationship with woody dicotyledons than with herbaceous model plants. Promoter cis-element analysis revealed enrichment of stress-responsive elements such as ABRE, MYB, and MYC, suggesting potential regulatory links to ABA-mediated drought signalling. Transcriptomic profiling showed that *GhEP1* genes are predominantly expressed in roots, stems, and floral organs rather than leaves, with *GhEP1-8* highly expressed in petals and stamens. Under drought stress, *GhEP1-2*, *GhEP1-7*, and *GhEP1-8* were significantly upregulated in roots, while *GhEP1-3* was induced in leaves, indicating tissue-specific regulatory divergence. qRT-PCR further confirmed that most *GhEP1* members were significantly upregulated in roots following drought treatment. Collectively, these findings provide a comprehensive framework for understanding the *GhEP1* gene family in cotton and identify *GhEP1-2*, *GhEP1-7*, and *GhEP1-8* as candidate genes that warrant further functional validation. Their exact roles in drought tolerance remain to be confirmed using stable transgenic lines and VIGS experiments. However, their exact roles in drought tolerance remain to be validated using stable transgenic lines, and the current findings should be considered as a foundation for future functional studies and molecular breeding of drought-tolerant cotton.

## Figures and Tables

**Figure 1:**
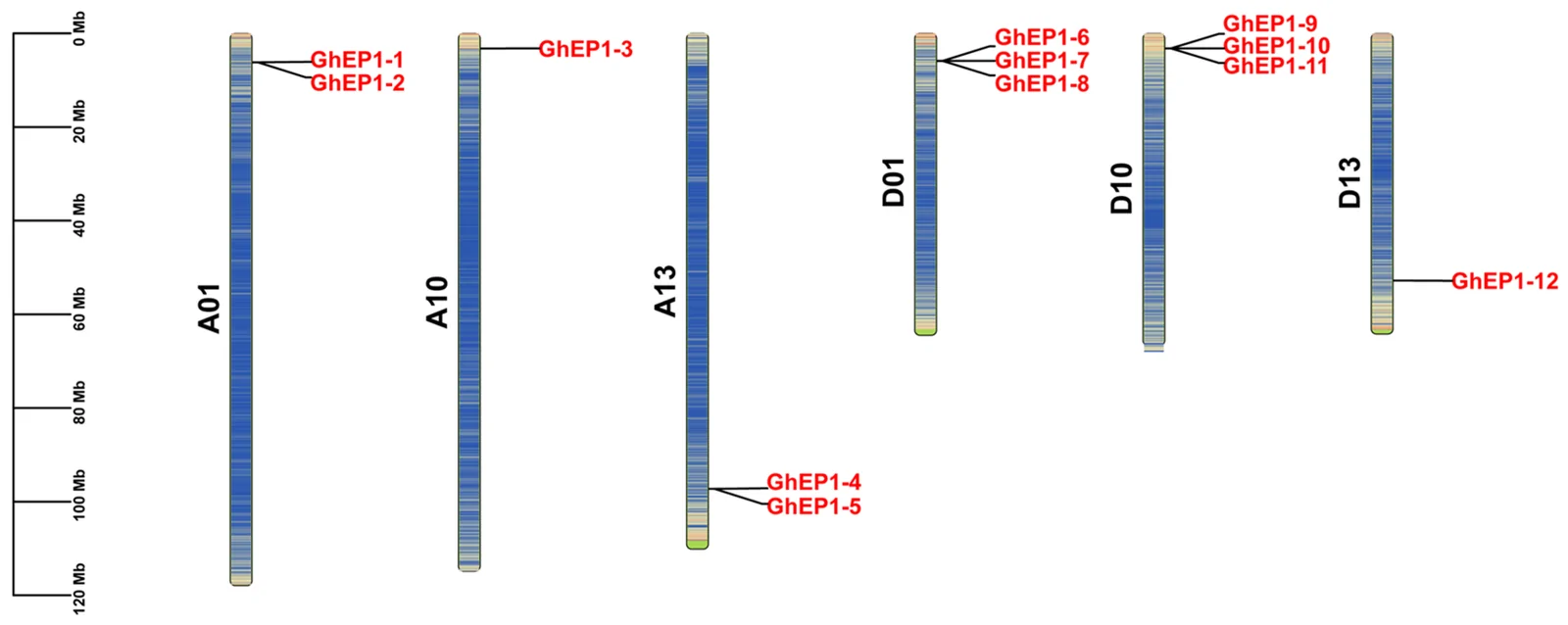
Chromosomal localisation of the *GhEP1* gene family in *G. hirsutum*. The black numbers represent the chromosome numbers. The red gene names to the right of the chromosomes correspond to the approximate locations of the genes. The different colours on the chromosomes indicate the abundance of genes on each chromosome.

**Figure 2:**
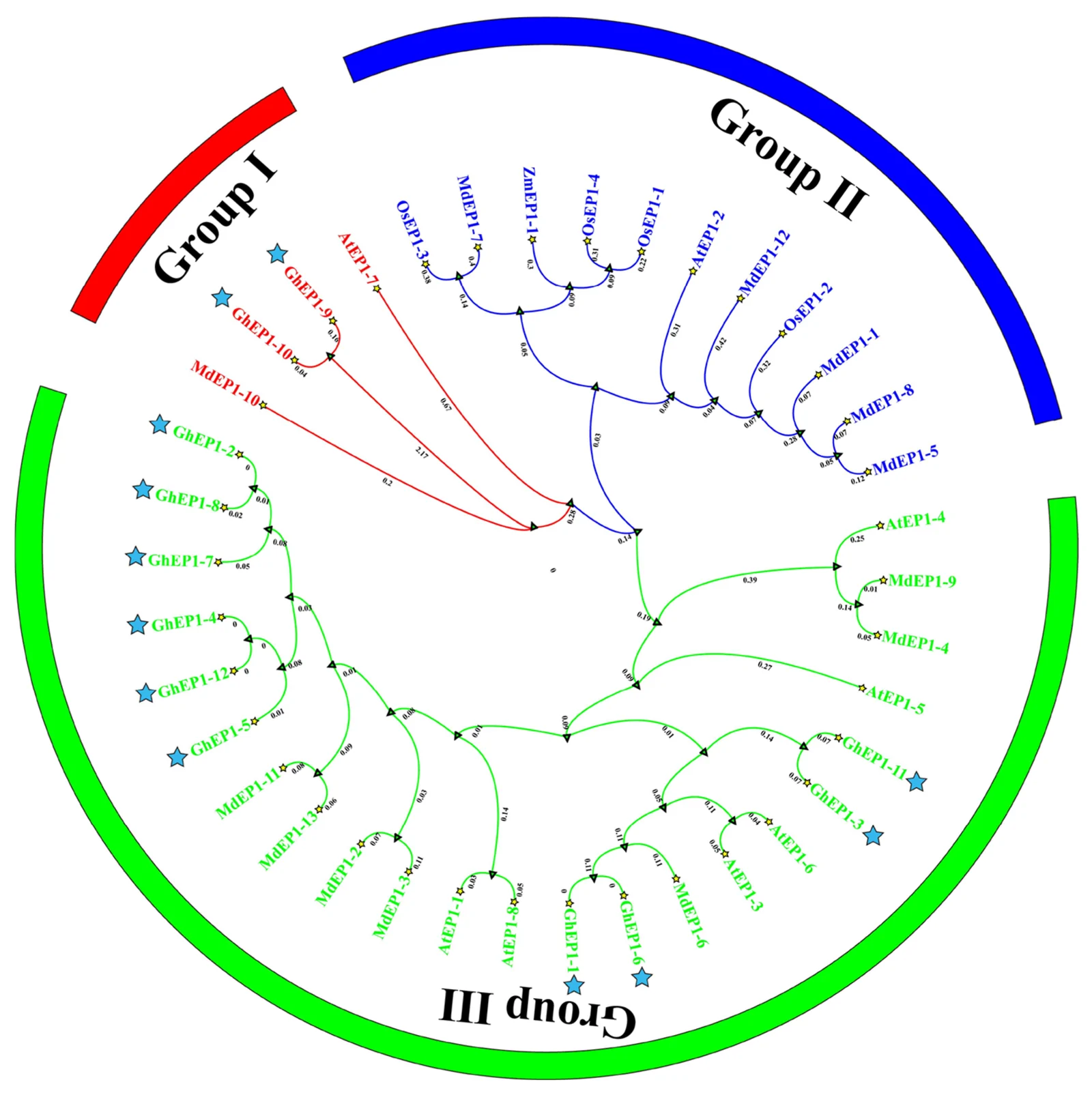
Phylogenetic tree of the GhEP1 protein family in *G. hirsutum*. Evolutionary and phylogenetic analysis of the *EP1* gene family. Evolutionary and phylogenetic analysis of the *EP1* gene family across five species: *AtEP1s* (*A. thaliana*), *ZmEP1s* (*Z. mays*), *MdEP1s* (*M. domestica*), *OsEP1s* (*O. sativa*) and *GhEP1s* (*G. hirsutum*), categorised into three subgroups based on different colours. Bootstrap values (1000 replicates) are shown at branch nodes; asterisks indicate bootstrap support > 70%.

**Figure 3:**
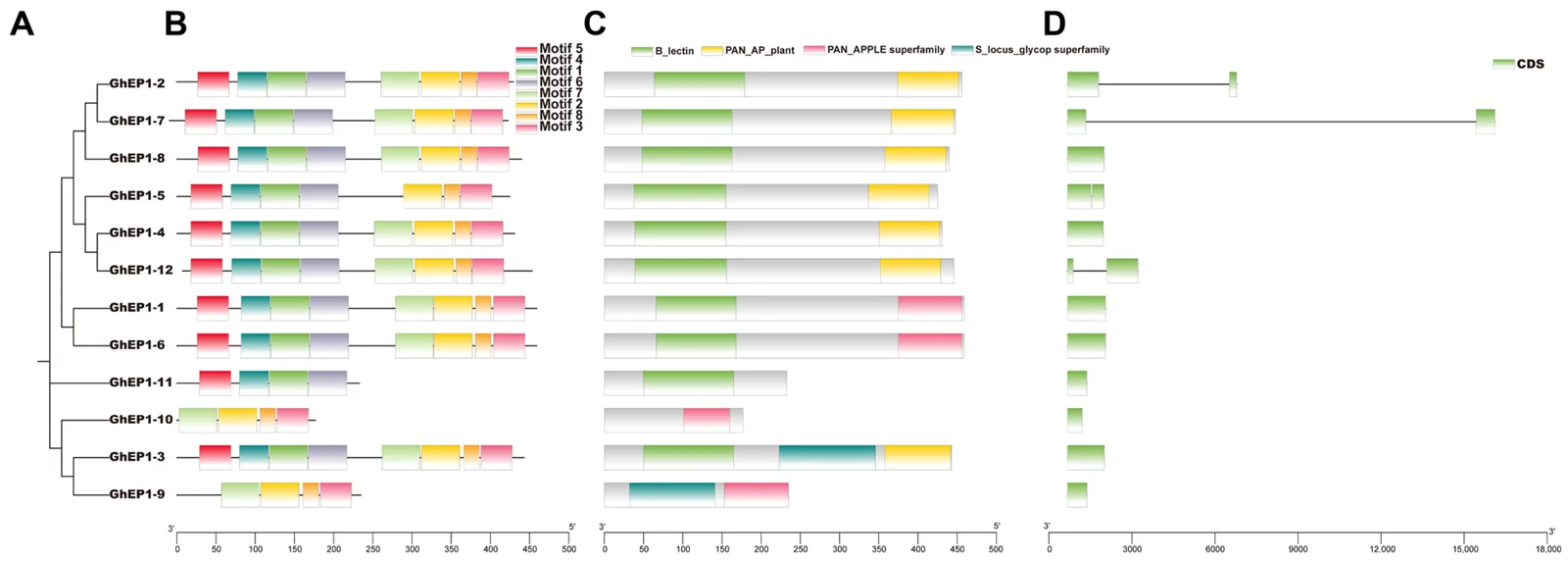
Analysis of the phylogenetic tree, motifs, conserved domains and gene structure of the *GhEP1* gene family in *G. hirsutum*. (**A**) Phylogenetic tree of the *GhEP1* gene family in *G. hirsutum*; (**B**) motif analysis, with each motif represented by a square of a different colour; (**C**) analysis of conserved domains, with different colours representing different domains; (**D**) gene structure analysis, with exons represented by green rectangles and introns by black lines.

**Figure 4:**
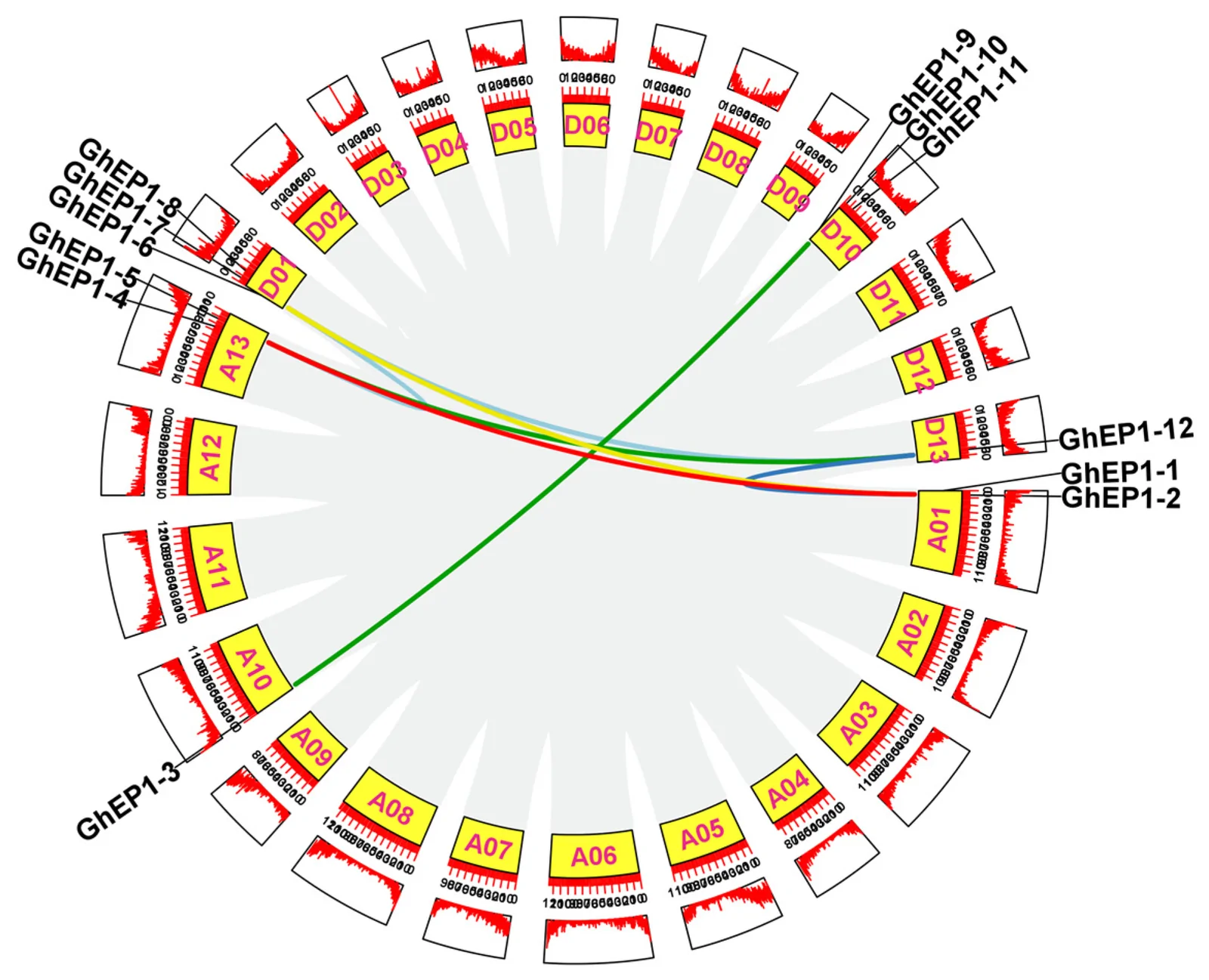
Intra-species colinearity analysis of the *GhEP1* gene family in *G. hirsutum*. Yellow sectors of varying sizes represent chromosomes of different sizes, whilst the red numbers denote chromosome IDs. The grey lines in the background represent colinearity blocks in the *G. hirsutum* genome, and the lines of different colours represent homologous *GhEP1* gene pairs. The white sectors represent gene abundance on the chromosomes.

**Figure 5:**
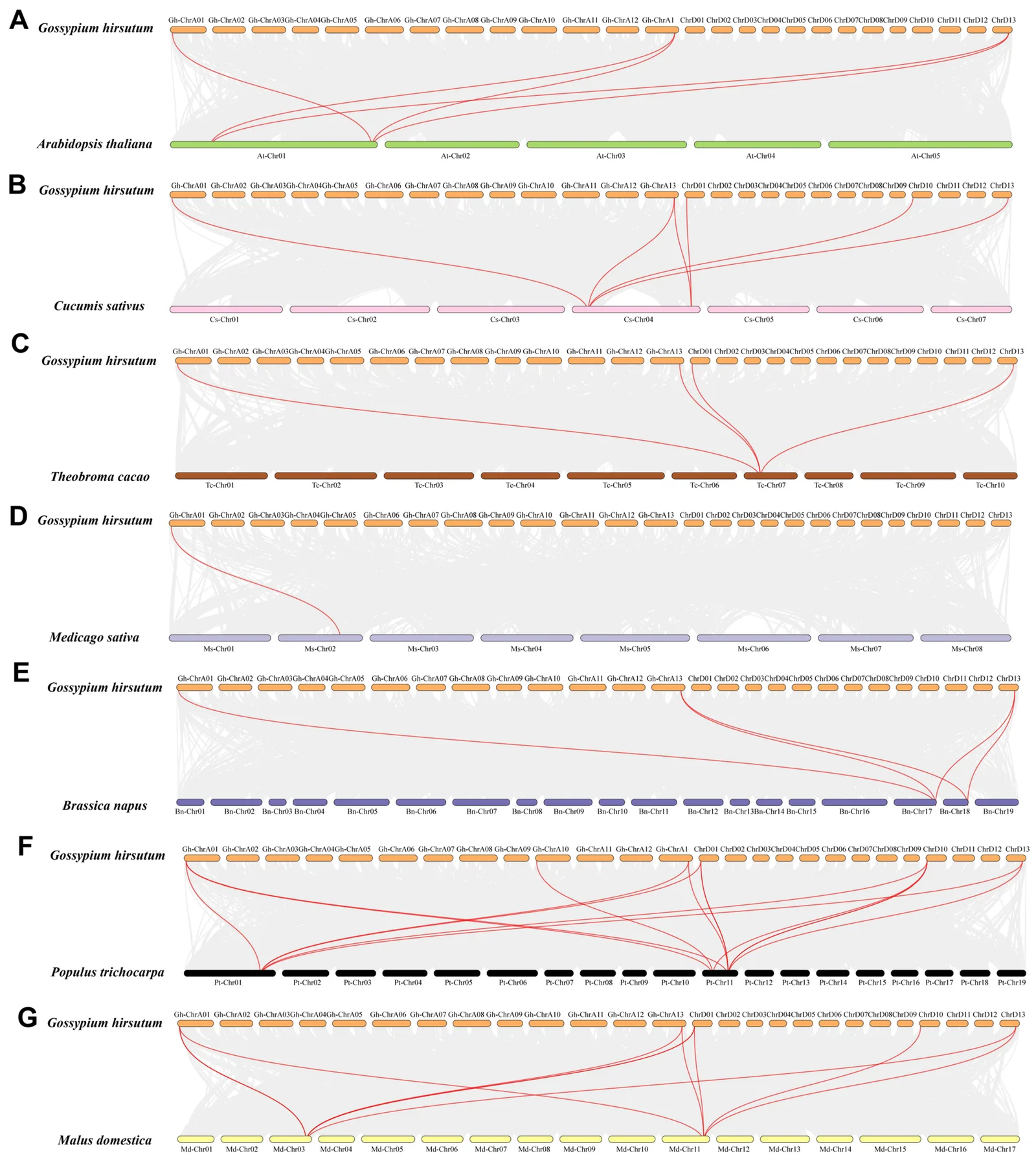
Analysis of colinearity between the *GhEP1* gene family of *G. hirsutum* and those of seven other species. The grey lines in the background represent colinearity blocks between the *G. hirsutum* genome and those of the other species, whilst the red lines indicate gene pairs within the *EP1* gene family. (**A**): *A. thaliana*; (**B**): *C. sativus*; (**C**): *T. cacao*; (**D**): *M. sativa*; (**E**): *B. napus*; (**F**): *P. trichocarpa*; (**G**): *M. domestica*.

**Figure 6:**
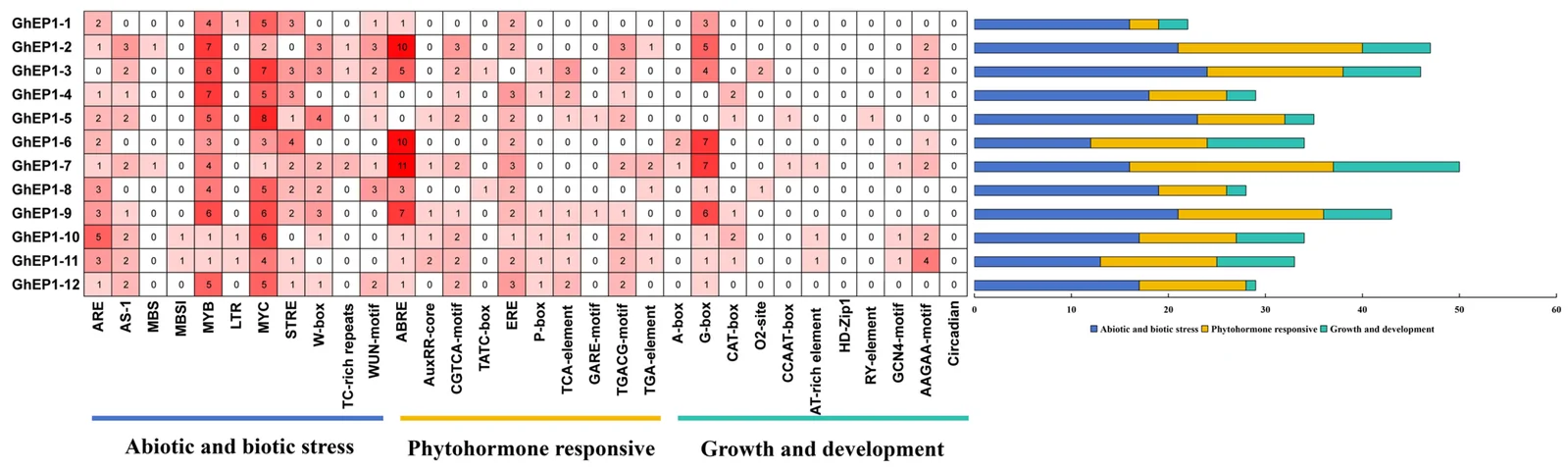
Analysis of cis-acting elements in the *GhEP1* gene family. The red gradient rectangles and the numbers within them in the heatmap indicate the number of cis-acting elements. The three colour-coded bar charts show the number of cis-acting elements with three different functions; these cis-acting elements in the promoter region are categorised into the following three groups based on their function: abiotic and biotic stress, phytohormone responsive, and growth and development.

**Figure 7:**
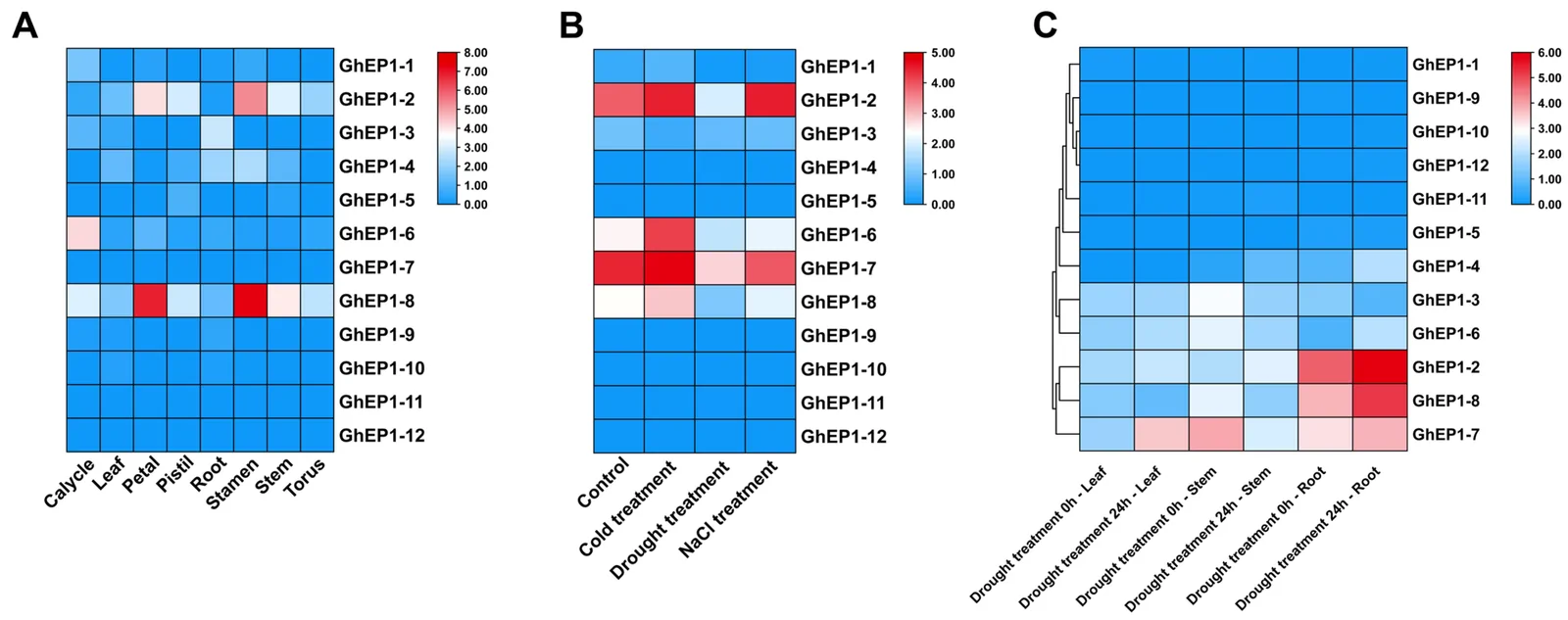
Heatmaps of *GhEP1* gene family expression profiles in FPKM from different tissues and organs of *G. hirsutum*. (**A**) Transcriptomic expression (FPKM) across eight tissues and organs. (**B**) Transcriptomic expression following cold, drought and NaCl stress treatments, data are from leaf tissues. (**C**) Transcriptome data under drought stress in different tissue treatments and at different times after treatments. Red, white and blue represent high, medium and low expression levels, respectively.

**Figure 8:**
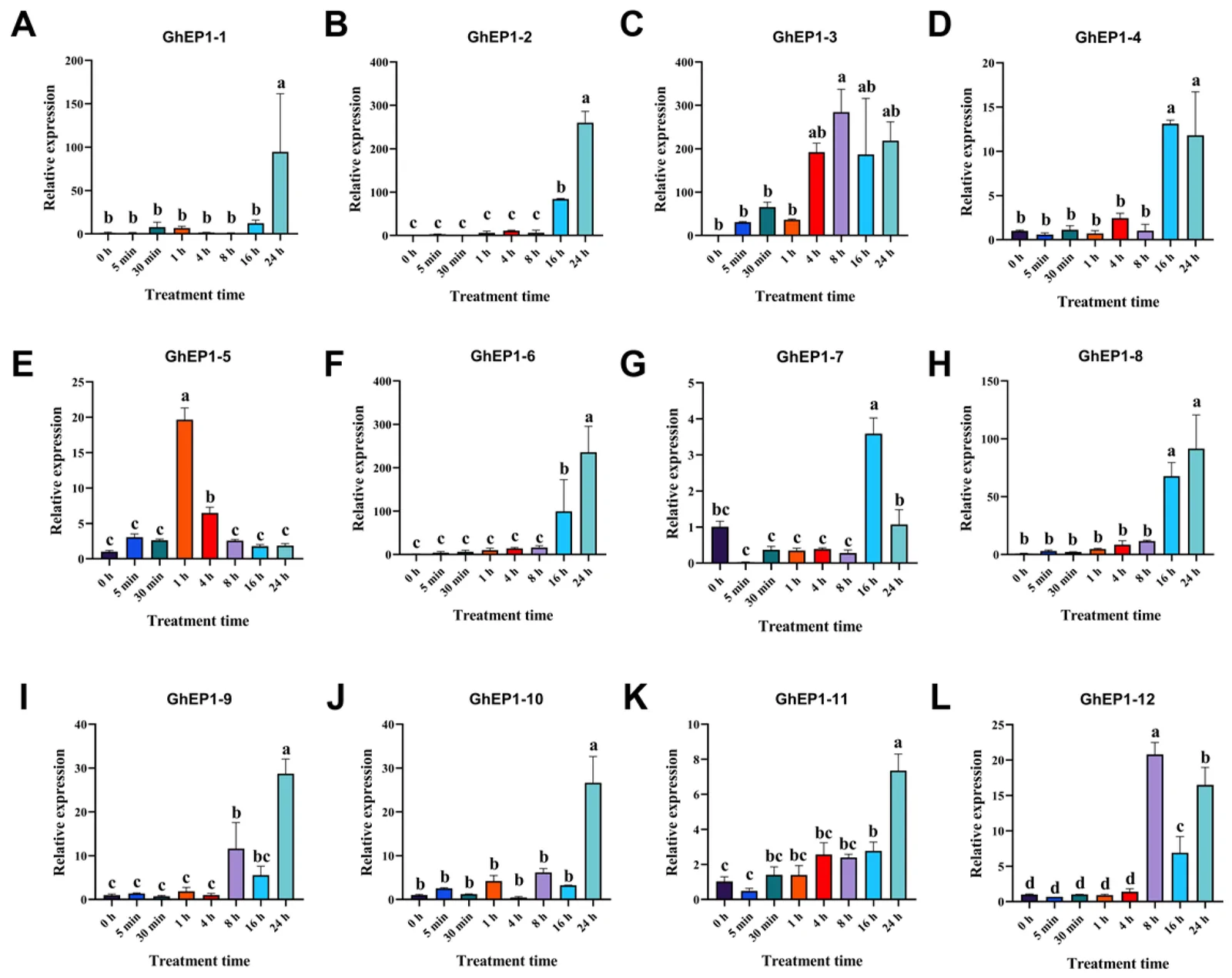
qRT-PCR analysis of the expression of members of the *GhEP1* gene family in roots following drought stress of varying durations. Figures (**A**–**L**) show the relative expression levels of *GhEP1-1* to *GhEP1-12*, respectively. Data are presented as ‘mean ± standard deviation’ (*n* = 3). Experimental data were analysed using the Bonferroni test; different letters on the bar charts indicate statistically significant differences between groups (*p* < 0.05).

**Figure 9:**
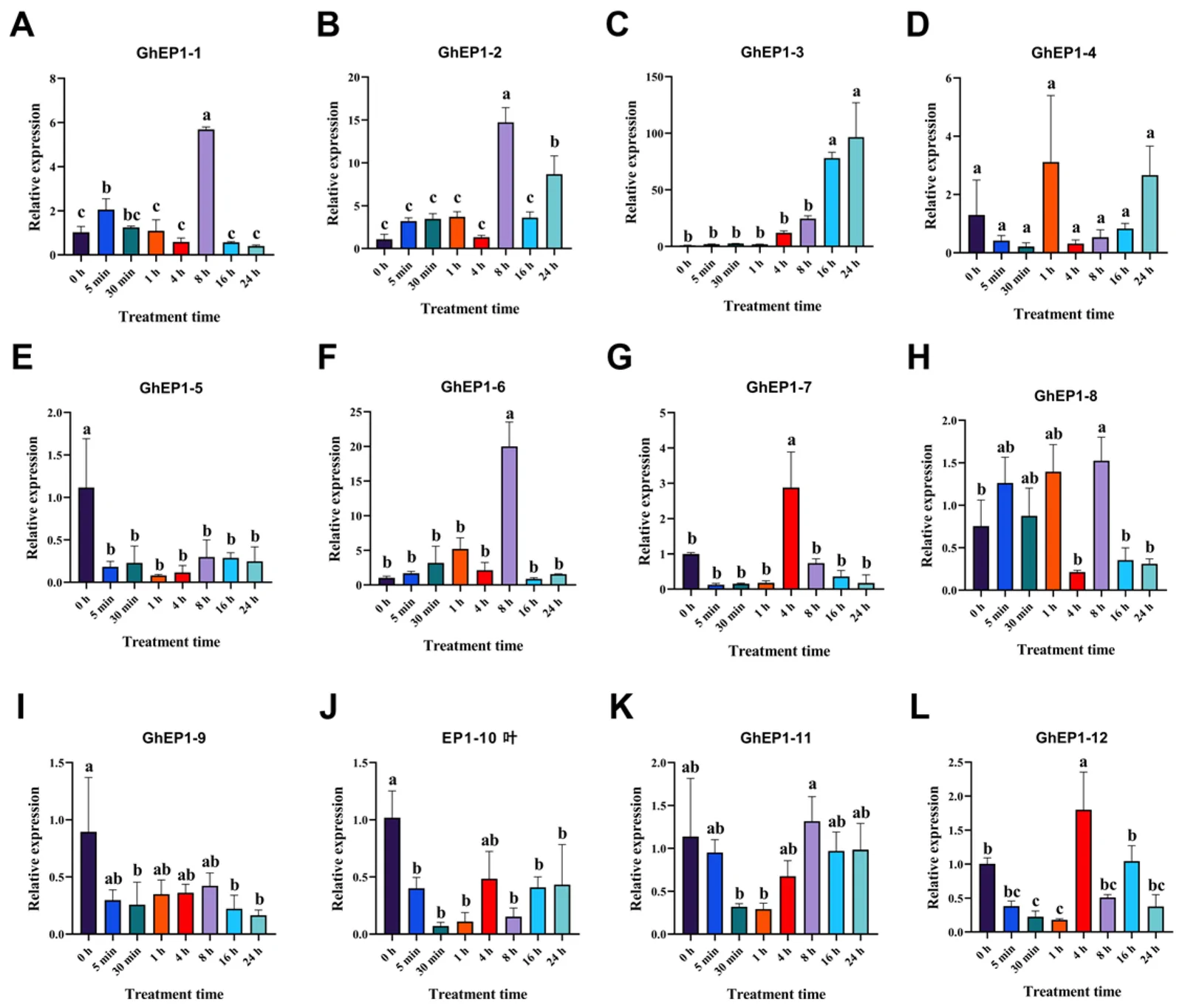
qRT-PCR analysis of the expression of members of the *GhEP1* gene family in leaves following drought stress of varying durations. Figures (**A**–**L**) show the relative expression levels of *GhEP1-1* to *GhEP1-12*, respectively. Data are presented as ‘mean ± standard deviation’ (*n* = 3). Experimental data were analysed using the Bonferroni test; different letters on the bar charts indicate statistically significant differences between groups (*p* < 0.05).

## Data Availability

All data supporting the conclusions of this paper are provided in the article and Supplementary Materials. Genome sequence data for all species are available in the Phytozome database (https://phytozome-next.jgi.doe.gov/, accessed on 20 November 2025) and COTTONOMICS database (http://cotton.zju.edu.cn/index.htm, accessed on 13 November 2025). Publicly available RNA-seq data are available on the COTTONOMICS database.

## Supplementary Materials

The following supporting information can be downloaded at: https://www.mdpi.com/article/10.3390/ijms27209303/s1.
