# Supplementary material for: Genome-Wide Identification and Expression Analysis of the S-Locus-Specific Glycoprotein/EP1 Gene Family in Cotton (Gossypium hirsutum L.)
Source: Int J Mol Sci. 2026 Oct 20;27(20):9303. doi: 10.3390/ijms27209303 (PMC13704920; doi:10.3390/ijms27209303)
Supplement: Supplementary file 1 [file ijms-27-09303-s001.zip › Figure S1-S2.pdf]

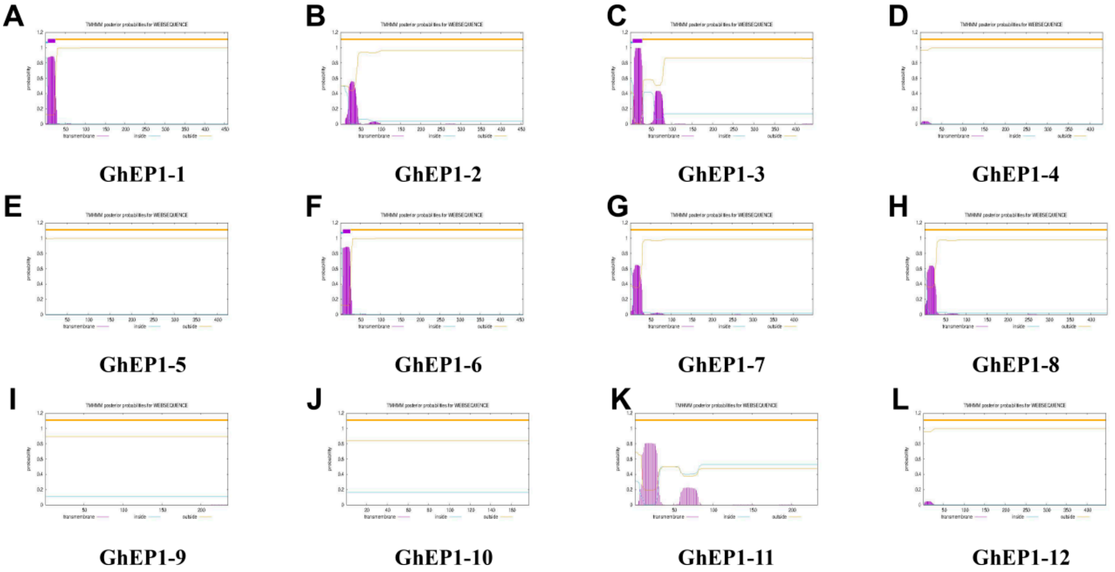

Figure S1. Prediction of the transmembrane helices of the 12 GhEP1 proteins in *G. hirsutum*. The protein identifiers *GhEP1-1* through *GhEP1-12* are shown below. In each protein sequence, the predicted transmembrane helices are highlighted by shaded regions (purple).

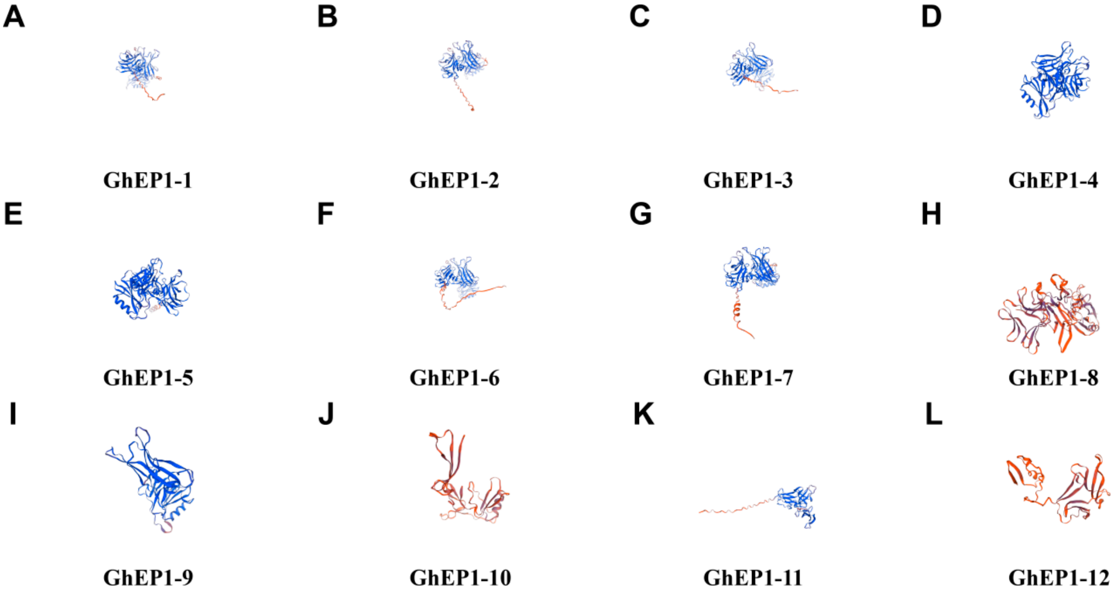

Figure S2. Predicted three-dimensional structures of 12 GhEP1 proteins in *G. hirsutum*. The homology models were generated using the SWISS-MODEL server. Panels A–L correspond to *GhEP1-1* through *GhEP1-12*. α-helices, β-strands, and loops are highlighted in different colors, and all models are rendered to the same scale.
